# Epidemiology and Diagnosis of Post-Thrombotic Syndrome: Qualitative Synthesis with a Systematic Review

**DOI:** 10.3390/jcm12185896

**Published:** 2023-09-11

**Authors:** Jitendra Mangwani, Veronica Roberts, Odei Shannak, Pip Divall, Ananth Srinivasan, Joseph Dias

**Affiliations:** 1 Academic Team of Musculoskeletal Surgery, University Hospitals of Leicester NHS Trust, Leicester LE1 5WW, UK; jitendra.mangwani@uhl-tr.nhs.uk (J.M.); pip.divall@uhl-tr.nhs.uk (P.D.); jd96@leicester.ac.uk (J.D.); 2 Department of Trauma and Orthopaedics, Southern Health and Social Care Trust, Belfast BT63 5QQ, UK; mrspowelly@icloud.com; 3 Department of Trauma and Orthopaedics, Northampton General Hospital NHS Trust, Northampton NN1 5BD, UK; odei.shannak1@nhs.net

**Keywords:** post-thrombotic syndrome, diagnosis, assessment, grading, incidence

## Abstract

***Background***: Post-thrombotic syndrome (PTS) is a common and debilitating sequela of lower limb deep vein thrombosis (DVT). There is significant heterogeneity in reported PTS incidence due to lack of standardised diagnostic criteria. This review aimed to develop diagnostic criteria for PTS and subsequently refine the reported incidence and severity. ***Methods***: PRISMA principles were followed; however, the review was not registered. The Cochrane CENTRAL database, MEDLINE, Embase, the NHS NICE Healthcare Databases Advanced Search interface, and trial registers including isrctn.com and clinicaltrials.gov were searched for studies addressing areas of interest (PTS definition, epidemiology, assessment). An experienced Clinical Librarian undertook the systematic searches, and two independent reviewers agreed on the relevance of the papers. Conflicts were resolved through panel review. Evidence quality was assessed using a modified Coleman scoring system and weighted according to their relevance to the aforementioned areas of interest. ***Results***: A total of 339 abstracts were retrieved. A total of 33 full-text papers were included in this review. Following qualitative analysis, four criteria were proposed to define PTS: (1) a proven thrombotic event on radiological assessment; (2) a minimum 24-month follow-up period after an index DVT; (3) assessment with a validated score; and (4) evidence of progression of venous insufficiency from baseline. Four papers conformed to our PTS definition criteria, and the incidence of mild to moderate PTS ranged from 7 to 36%. On reviewing the studies which utilised the recommended Villalta scale, PTS incidence narrowed further to 23–36%. Incidence and severity reached a plateau at 24 months. ***Conclusions***: Four diagnostic criteria were developed from qualitative synthesis. When these criteria were applied to the literature, the range of reported PTS incidence narrowed. These four criteria may standardise PTS diagnosis in future studies, facilitating the pooling of data for meta-analysis and synthesis of higher levels of evidence.

## 1. Introduction

Post-thrombotic syndrome (PTS) is the most prevalent sequela of deep vein thromboses (DVT) [1,2]. Valvular compromise and obstruction gives rise to chronic venous insufficiency and is responsible for the constellation of PTS symptoms and signs. Patients may experience pain, cramps, heaviness of the limb, leg swelling, pruritus, paraesthesia, and activity-related pain. Clinical signs on examination include limb oedema, induration, venous ectasia/varicose veins, hyperpigmentation, venous eczema, and, in the most severe of cases, intractable ulceration [1,2]. PTS can be a disabling condition which may threaten the affected limb while significantly reducing quality of life and posing an economic burden to society [1,2].

The latency period from an index DVT to PTS development is generally reported to be within two years; however, studies describe increasing cumulative incidence over the subsequent two decades [2,3,4]. There is a significant range in reported incidence (20–63%) and the spectrum of disease severity due to a lack of standardised diagnostic criteria [3,4,5,6,7,8]. Despite the well-documented two-year latency period, the follow-up in the literature is often too short. PTS scoring systems are available to aid diagnosis. However, the available systems are composed of a heterogeneous distribution in scoring items and hence are not directly comparable. The validated Villalta scale and its variations including the patient-reported Villalta (PRV) scale account for both symptoms and findings from clinical examination [9]. The Clinical–Etiology–Anatomy–Pathophysiology (CEAP) classification differs from the Villalta scale by weighting PTS components identified by clinical examination alone [10]. These inconsistencies in assessing and diagnosing PTS compromise accurate epidemiological study, inter-study comparisons, meta-analyses, and the generation of higher levels of evidence [11].

Despite receiving more attention in recent years, there remains a paucity of high-quality epidemiological data. The primary aim of this systematic review and qualitative synthesis is to appraise the available literature and propose a standardised way of diagnosing PTS. The authors aim to report PTS incidence with the assessment of severity utilising these proposed criteria.

## 2. Methods

This systematic review was performed in accordance with the Preferred Reporting Items for Systematic Reviews and Meta-Analyses (PRISMA) reporting guidelines for conducting a meta-analysis [12].

### 2.1. Inclusion Criteria

- Human study participants: adult patients (18 or older), both sexes, all ethnicities, all nationalities.
- Consensus statements, recommendations, standards of practice.
- Original articles relevant to post-thrombotic syndrome (or post-phlebitic syndrome, venous stress disorder, and venous thrombosis complication/sequelae) secondary to DVT reporting incidence and method of disease assessment.
- English-language publications.

### 2.2. Exclusion Criteria

- Conference abstracts, case reports, studies with fewer than 10 patients.

### 2.3. Study Outcomes

The primary outcome measure was to propose diagnostic criteria for PTS. Utilising the proposed criteria, the panel aimed to establish incidence with assessment of severity.

### 2.4. Literature Search Strategy

Cochrane CENTRAL database, MEDLINE, EMBASE, NHS NICE Healthcare Databases Advanced Search interface, and trial registers including isrctn.com and clinicaltrials.gov were searched by an experienced Clinical Librarian (PD) from the databases’ inception to August 2023. The searches were performed using exploded medical subject headings and corresponding appropriate terms (Supplementary Materials). The references of relevant articles were manually searched to identify additional eligible studies.

### 2.5. Study Selection

Two senior authors (OS and VR) independently assessed all abstracts and recorded study characteristics on a pre-agreed Excel (Microsoft) proforma. Full versions of the articles were obtained if they conformed to the inclusion/exclusion criteria and were deemed relevant to PTS assessment and diagnosis. Disagreements in the selection of studies were resolved by study panel discussion and consensus.

### 2.6. Data Extraction and Critical Appraisal

Data were extracted to a pre-designed proforma on Microsoft Excel and included study design (consensus statements, original research articles), author, year, journal of publication, level of evidence, sample size, method of initial DVT diagnosis, duration follow-up, PTS scoring methodology, and PTS incidence and severity. Each article was weighted by its methodology, using the modified Coleman scoring system (Supplementary Materials, Table S1) [13], which takes into account the study design and quality of reported outcomes, allowing effective commentary on the overall study quality [13]. Each article was then weighted by its relevance to a particular area of interest in the systematic review (incidence, assessment, and severity) using a further modification of the Coleman scoring system (Supplementary Materials, Table S2).

### 2.7. Data Synthesis and Statistical Analysis

Qualitative synthesis was undertaken using NVivo-10 software (QSR International, UK) to identify themes for PTS diagnostic criteria. In case of substantial heterogeneity in study design, a narrative approach was used to analyse the data.

### 2.8. Patient and Public Involvement

Patients and the public were not involved in the design, implementation, reporting, or dissemination plans of our research.

## 3. Results

### 3.1. Search Strategy

**Figure 1:**
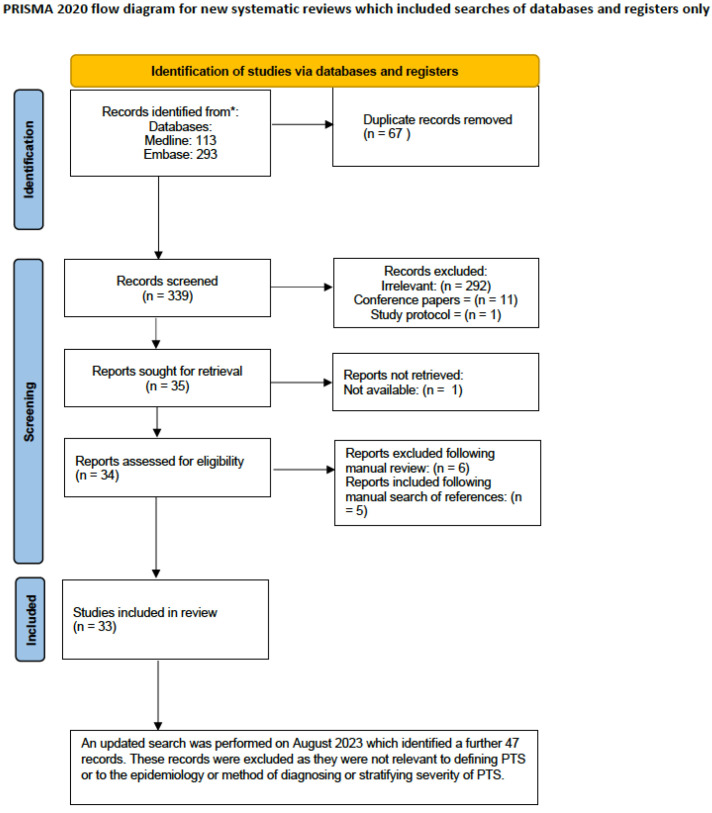
PRISMA flow diagram. A total of 339 original abstracts were retrieved through a search of CENTRAL, MEDLINE, Embase, and trial registers. The search was updated in August 2023.

The details of the search and exclusion criteria are displayed in the flow diagram (Figure 1). The initial search strategy was conducted in January 2022, which yielded 339 original abstracts. The search was repeated in August 2023, and a further 47 abstracts were retrieved. The two independent reviewers agreed on the inclusion of 41 abstracts. The original publications were obtained, and thirteen articles were excluded. Another five abstracts were identified from a manual search of reference lists.

### 3.2. Proposed Definition

Seven articles of sufficient methodological rigour were identified for qualitative analysis (Supplementary Materials, Table S3) [10,11,14,15,16,17,18]. Four criteria for PTS diagnosis were identified and further refined by the review panel.

1. A clear thrombotic aetiology must be demonstrated [11,14,15]. The index DVT should be proven by gold-standard radiological investigations such as proximal leg vein ultrasonography or contrast studies [19].
2. The affected and contralateral limbs should then be assessed with a validated scoring system, ideally the Villalta scale or the Clinical–Etiology–Anatomy–Pathophysiology (CEAP) classification [11,20,21].
3. Patients should be followed up prospectively at pre-defined time intervals (every three to six months) for a minimum period of two years in order to establish PTS diagnosis. Diagnosis within three months of the index DVT should be avoided as the acute disease process may exhibit confounding signs and symptoms [11].
4. PTS manifestations during the follow-up period should be confirmed as new or a significant worsening from baseline. This emphasises the importance of initial assessment and documentation of bilateral lower limb venous drainage at the time of the index DVT [7,22,23,24,25,26,27,28].

These four diagnostic criteria formed the basis of analysis for the subsequent areas of interest in this systematic review.

### 3.3. PTS Assessment and Severity Grading

Twenty-one papers scored a minimum of 60 out of 100 on the Coleman score to review the methodology of PTS assessment [7,11,20,21,23,24,25,26,27,28,29,30,31,32,33,34,35,36,37,38,39] (Supplementary Materials). Nineteen of these papers were of sufficient quality to review the method of grading PTS severity (Supplementary Materials, Table S3). Seven different scoring systems were used (Table 1), with the majority of studies utilising either the Villalta scale [11,21,26,27,28,33,34,35,37,38] or CEAP classification [20,23,29,30,31,36]. The remaining five scores were each used once: Brandjes [7]; Kakkar–Lawrence [16]; Ginsberg [25]; Widmer [32]; and Browse [35]. Two articles [21,35] utilised the VEINES-QOL questionnaire, a health and quality of life assessment tool, alongside the Villalta score.

The various systems share common items (Table 1) which address subjective and/or objective findings. The Widmer and Browse scores consist of objective findings only. The CEAP classification was first developed to diagnose and stratify the severity of general chronic venous disease. CEAP scores patients dichotomously as either symptomatic or asymptomatic and does not discern specific symptoms. The CEAP objective items grade PTS from Class 0 to 6. Classes 2 and 3 correlate with mild to moderate PTS while Classes 4 to 6 are considered severe. The Villalta score, specifically developed for PTS, uses five symptoms and six clinical signs, each scored 0–3 for severity. A score between 5 and 9 indicates mild PTS; between 10 and 14 indicates moderate disease; and any score greater than 15 or the presence of an ulcer indicates severe disease.

### 3.4. Incidence

Of the 33 papers, 24 were found to be of sufficient quality, scoring a minimum of 70 out of 100 in the modified Coleman score [7,16,20,22,23,24,25,26,27,28,30,31,32,33,34,35,36,37,38,40,41,42,43,44] (Supplementary Materials, Table S3). Three studies were excluded as PTS incidence was not reported [30,31,41]. A further four were excluded as the duration of follow-up was less than 24 months from the index DVT [40,42,43,44]. The remaining 17 papers used a scoring system for clinical assessment, with 11 using either Villalta or CEAP scores. Six studies using the unvalidated Browse assessment tool [22,24], Brandjes [7], Kakkar–Lawrence [16], Widmer [32], or Ginsberg [25] scores were excluded in the incidence analysis. A further seven studies were excluded as an initial assessment of pre-existing chronic venous insufficiency was not reported [20,26,33,34,35,36,37].

**Figure 2:**
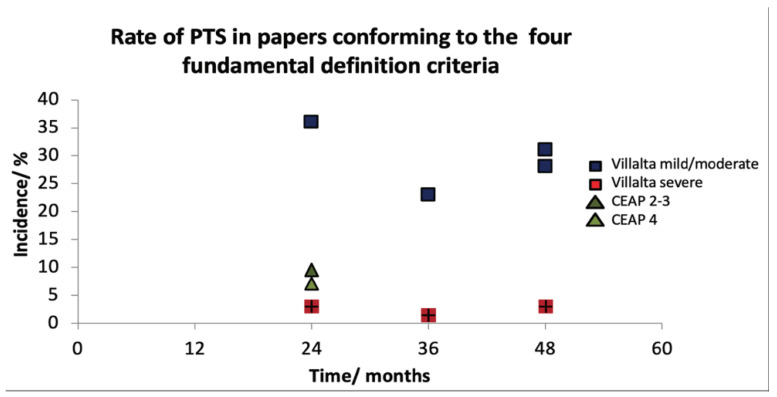
A timeline graph demonstrating that incidence and severity becomes established by 24 months following index DVT.

**Figure 3:**
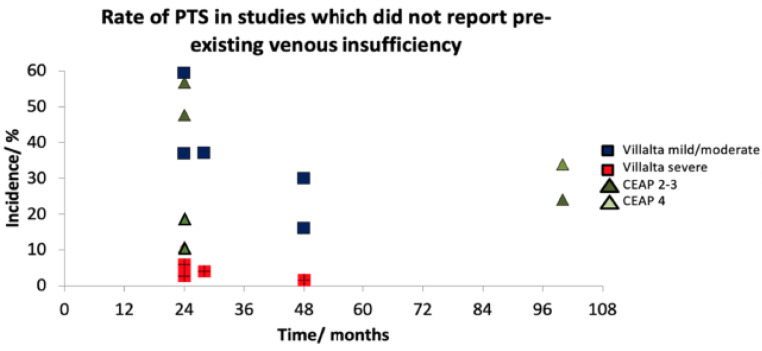
A timeline graph demonstrating a higher rate of PTS when pre-existing venous insufficiency was not assessed. However, PTS incidence and severity remains established at 24 months following index DVT.

Four papers conformed to our PTS definition criteria [23,27,28,38]. The rate of mild or moderate PTS in patients was 7–36% (Table 2). The rate of severe PTS in these patients was 1.5–3%. PTS incidence narrowed (23–36%) when studies using CEAP scores were removed. When the criterion of assessment of pre-existing venous insufficiency was omitted, a further seven papers could be included [20,26,33,34,35,36,37]. The resulting rate of PTS increased, and the range widened to 16–59% for mild or moderate but remained similar for severe PTS (1.6-5.9%) (Table 3). Incidence remained unaffected by time after 24 months (Figure 2 and Figure 3).

## 4. Discussion

Post-thrombotic syndrome is associated with increased biopsychosocial morbidity and healthcare costs [1]. Heterogeneity in diagnostic criteria has compromised PTS research, making epidemiological study, meta-analysis of proportions, and inter-study comparison difficult. Four standardised diagnostic criteria for PTS definition are proposed in this study:

○ A radiologically proven index DVT.
○ A minimum 24-month follow-up period with regular interval assessment.
○ Assessment with a validated scoring system (ideally the PTS-specific Villalta scale).
○ Documentation of baseline venous drainage with demonstration of disease progression.

PTS incidence narrowed from 20–63% to 23–36% on application of our proposed diagnostic criteria. 

The diagnosis of the initial DVT is widely agreed in the PTS literature [11,14,15,16,17,18], with current NICE guidance recommending an urgent ultrasound scan of the affected limb [19]. A proximal location or extensive thrombus may confer higher PTS risk, and hence it is of clinical value to record the anatomical location and thrombus extent during diagnostic imaging [28]. 

Baseline venous drainage may be difficult to establish with confidence due to the confounding effect of the obstructing index DVT. Ideally, this assessment should reflect the venous drainage of the limb prior to the occurrence of the index DVT. As this is unrealistic, the authors recommend a pragmatic approach where the state of venous drainage of both the affected and contralateral limbs are assessed and documented at the time of DVT diagnosis. To mitigate the confounding effects of the index DVT, the authors recommend that the contralateral limb be used cautiously as a proxy for the prior state of venous drainage of the affected limb. Further deterioration may then be determined at prospective follow-up. The limitations of this approach include the lack of available literature and presence of prior contralateral venous disease.

Future studies with PTS diagnosis as an endpoint should review patients for a minimum period of 24 months. PTS incidence at this time period seems to plateau and disease severity becomes established [23,27,28,38]. It is not practical or cost-efficient to follow all patients with a DVT to assess the progression of venous insufficiency or PTS in clinical practice; hence, the authors advocate this minimum follow-up duration for the research setting. The benefit of three- to six-month periodic evaluation may be useful in establishing the latency period for academic purposes and requires future research. The authors accept that the assessment of progressive venous insufficiency may be difficult in instances where the thrombus fails to recanalise and the deep venous system remains obstructed. Bicuspid venous valves are responsible for venous competence and are concentrated peripherally at the calf level where hydrostatic pressure is greatest. Reflux at the level of the valve cusps may be noted on ultrasound imaging in venous insufficiency and can vary from minor leaks to profound incompetence. Valsalva, simulated Valsalva, and manual augmentation can assist in demonstrating valvular incompetence, and colour Doppler ultrasonography assists the operator in identifying the exact locations of refluxing valves [45]. These pathological changes may be periodically observed during the development of PTS [45]. Future work should focus on objective confirmation of venous insufficiency (reflux or valvular incompetence) at the time of PTS diagnosis by means of an investigation such as Doppler ultrasound or photoplethysmography and should be carried out periodically to document worsening of the disease [41].

Studies in the literature rely purely on scoring systems to diagnose PTS. Despite similarities between the systems, there is marked variation in reported PTS incidence. This is exemplified by papers using validated scores alone (7–36%) [23,27,28,38]. Systems that relied predominantly on examination findings rather than symptoms such as CEAP unsurprisingly diagnosed fewer cases [23,46]. PTS is a constellation of patient-reported symptoms and clinical signs, and hence the ideal scoring system should consider both. The International Society on Thrombosis and Haemostasis and the Scientific and Standardisation Committee endorse the Villalta scale for diagnosing PTS and grading disease severity [11]. The Villalta scale has demonstrated excellent validity, inter-rater reliability, sensitivity, and acceptability following systematic study [17]. Other reported advantages include binary, categorical, and continuous outcome measures that may be pooled for meta-analysis. The concern with utilising the Villalta scale alone is that nonspecific symptoms (pain, paraesthesia) and signs (oedema) achieving the threshold for PTS diagnosis (≥ 5) could have an altogether different aetiology to PTS. Thus, the panel advocate for the Villalta scale to be used in conjunction with the three aforementioned diagnostic criteria. Ng et al. (2022) recently validated the patient-reported Villalta scale (PRV) which may significantly reduce the burden on resources and clinical workload during patient follow-up [47]. The kappa value and agreement were 0.60 and 81%, respectively. However, the reported PTS rate was much higher with PRV (42%) compared with the Villalta scale (33%), and agreement between the scores tended to improve only when assessing PTS cases of greater severity [47]. PRV may be an acceptable and less-resource-demanding tool, but further work is necessary to narrow the variability in reported PTS. 

PTS confers significant associated biopsychosocial morbidity and risk to the limb. Therefore, patients developing venous insufficiency or diagnosed with PTS should be promptly referred to a local specialist vascular multidisciplinary team for review. Team members range from vascular specialist nurses to vascular surgeons who can advise on further management [48].

**Figure 4:**
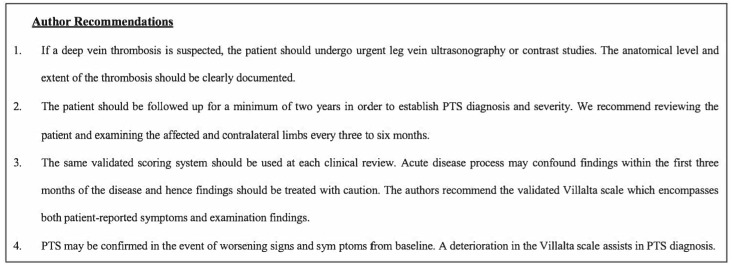
A summary of the four proposed diagnostic criteria.

PTS is a vast topic with the literature focusing on various aspects from epidemiology to prevention and interventions. We focused particularly on incidence, the method of diagnosis, and the stratification of disease severity, with the emphasis of developing diagnostic criteria. We are of the opinion that these four themes will form a standardised foundation for designing future studies investigating PTS prevention, intervention strategies, and subsequent meta-analyses (Figure 4). Future work should focus on raising awareness of PTS as a sequela of DVT. Furthermore, the four themes require further review by professional bodies and multidisciplinary stakeholders prior to use in research and clinical settings. 

## Figures and Tables

**Table 1:** A summary of available scores evaluating post-thrombotic syndrome with consideration of subjective and objective components. Villalta, CEAP, and Brandjes tools categorise severity.

	Villalta (Mild: 5–9, Moderate: 10–14, Severe: 15/Ulcer)	CEAP (Mild to Moderate: Class 2–3, Severe: Class 4–6)	Widmer	Browse (PTS = 4 or More)	Brandjes (Mild to Moderate ≤ 3, Severe ≥ 4)	Ginsberg
Subjective						
Symptomatic		Yes/No				
Pain	0–3					√
Cramps	0–3					
Heaviness	0–3				1	
Pruritus	0–3					
Paraesthesia	0–3					
Swelling					1	
Activities					1	
Pain (calf)—Spontaneous					1	
Pain (calf)—Stand/walk					1	√
Pain (thigh)—Spontaneous					1	
Pain (thigh)—Stand/walk					1	√
Objective						
Telangiectasia/flare	0–3	Class 1		2	1	
Varicose Veins		Class 2	Class 1	1	1	
Oedema	0–3	Class 3	Class 1–3	2		√
Eczema	0–3	Class 4	Class 3		1	
Hyperpigmentation	0–3	Class 4	Class 2	2	1	
Induration	0–3			4	1	
Healed ulcer		Class 5	Class 2			
Ulcer	Severe	Class 6	Class 3	5	4	
Pain on compression	0–3					
>1 cm calf circumference					1	
>1 cm ankle circumference					1	

**Table 2:** Rate and severity of PTS in papers conforming to the four fundamental definition criteria.

	Sample Size	Follow-Up (Months)	Score	Severity	n (%)
Schindler and Dalziel (2005) [23]	42	24	CEAP	Mild Moderate	3 (7%) 4 (9.5%)
Delluc et al. (2010) [27]	95	48	Villalta	Mild/Moderate Severe	27 (28%) 0
Hach-Wunderle et al. (2013) [28]	135	36	Villalta	Mild/Moderate Severe	31 (23%) 2 (1.5%)
Prandoni et al. (2004) [38]	166	24	Villalta	Mild/Moderate Severe	60 (36%) 5 (3%)
Prandoni et al. (2004) [38]	126 *	48	Villalta	Mild/Moderate Severe	40 (31%) 4 (3%)

* Prandoni et al., 2004 [38] 126 patients at 48 months follow-up.

**Table 3:** Rate and severity of PTS in papers that did not assess pre-existing venous insufficiency.

	Sample Size	Follow-Up (Months)	Score	Severity	n (%)
Haenen et al. (2002) [20]	86	12–24	CEAP	Mild Moderate	41 (47.6%) 16 (18.6%)
Mant et al. (2008) [26]	25	24–48	Villalta	Any	4 (16%)
Kahn et al. (2014) [33]	803	6–24	Villalta	Mild/Moderate Severe	297 (36.9%) 47 (5.9%)
Partsch et al. (2004) [34]	37	24	Villalta CEAP	Mild/Moderate Mild Moderate Severe	22 (59.4%) 21 (56.8%) 4 (10.8%) 1 (2.7%)
Kahn et al. (2005) [35]	145	13–43	Villalta	Mild/Moderate Severe	54 (37%) 6 (4%)
Saarinen et al. (2002) [36]	50	72–120	CEAP	Mild Moderate	12 (24%) 17 (34%)
Galanaud et al. (2012) [37]	137	6–48	Villalta	Mild/Moderate Severe	100 (29.9%) 6 (1.6%)

## Data Availability

Data is contained within the article and supplementary material. Other data available on request.

## Supplementary Materials

The following supporting information can be downloaded at: https://www.mdpi.com/article/10.3390/jcm12185896/s1. Section 1. PTS Search Strategy: Medline and Embase. Sections 2 & 3. Modified Colman Scoring. Table S1. The Agreed Modified Coleman for the Generality of the article. Table S2. The agreed Modified Coleman weighted for areas of study interest. Table S3. Modified Coleman score summary, consideration by areas of interest.
