# Supplementary material for: Epidemiology and Diagnosis of Post-Thrombotic Syndrome: Qualitative Synthesis with a Systematic Review
Source: J Clin Med. 2023 Sep 11;12(18):5896. doi: 10.3390/jcm12185896 (PMC10532000; doi:10.3390/jcm12185896)
Supplement: Supplementary file 1 [file jcm-12-05896-s001.zip › jcm-2519458-supplementary.pdf]

Journal of
Clinical Medicine

MDPI

Supplementary Materials

Section 1. PTS Search Strategy: Medline and Embase

Evidence Services | library.nhs.uk

Search History

1. MEDLINE; (("post thrombotic" OR postthrombotic OR post-thrombotic)).ti,ab; 1652 results.
2. MEDLINE; exp POSTPHLEBITIC SYNDROME/; 568 results.
3. MEDLINE; (("post phlebitic" OR postphlebitic OR post-phlebitic OR (post adj3 phlebitic)) AND syndrome).ti,ab; 398 results.
4. MEDLINE; (venous AND stress AND disorder).ti,ab; 58 results.
5. MEDLINE; (chronic AND venous AND (disorder OR disease)).ti,ab; 4566 results.
6. MEDLINE; exp VENOUS INSUFFICIENCY/; 6132 results.
7. MEDLINE; (chronic AND venous AND insufficiency).ti,ab; 3041 results.
8. MEDLINE; 1 OR 2 OR 3 OR 4 OR 5 OR 6 OR 7; 12025 results.
9. MEDLINE; (lower AND limb*).ti,ab; 46464 results.
10. MEDLINE; exp LOWER EXTREMITY/; 136897 results.
11. MEDLINE; (leg OR hip OR knee OR ankle OR foot).ti,ab; 316737 results.
12. MEDLINE; 9 OR 10 OR 11; 423071 results.
13. MEDLINE; (orthopedic* OR orthopaedic*).ti,ab; 57272 results.
14. MEDLINE; exp ORTHOPEDIC PROCEDURES/; 217300 results.
15. MEDLINE; arthroplast*.ti,ab; 38966 results.
16. MEDLINE; exp ARTHROPLASTY/; 43087 results.
17. MEDLINE; ((total (hip OR knee OR ankle) AND replacement) OR thr OR tkr OR tar).ti,ab; 41013 results.
18. MEDLINE; exp JOINT PROSTHESIS/; 36163 results.
19. MEDLINE; (joint AND replacement).ti,ab; 9372 results.
20. MEDLINE; 13 OR 14 OR 15 OR 16 OR 17 OR 18 OR 19; 313537 results.
21. MEDLINE; 8 AND 12 AND 20; 142 results.
22. EMBASE; (("post thrombotic" OR postthrombotic OR post-thrombotic)).ti,ab; 2293 results.
23. EMBASE; exp POSTTHROMBOSIS SYNDROME/; 1652 results.
24. EMBASE; (("post phlebitic" OR postphlebitic OR post-phlebitic OR (post adj3 phlebitic)) AND syndrome).ti,ab; 405 results.
25. EMBASE; (venous AND stress AND disorder).ti,ab; 100 results.
26. EMBASE; exp CHRONIC VEIN INSUFFICIENCY/; 2946 results.
27. EMBASE; (chronic AND (vein OR venous) AND insufficiency).ti,ab; 4494 results.
28. EMBASE; 22 OR 23 OR 24 OR 25 OR 26 OR 27; 8476 results.
29. EMBASE; (lower AND limb*).ti,ab; 58613 results.
30. EMBASE; exp LEG/; 161141 results.
31. EMBASE; (leg OR hip OR knee OR ankle OR foot).ti,ab; 362396 results.
32. EMBASE; (lower AND extremity).ti,ab; 31938 results.
33. EMBASE; 29 OR 30 OR 31 OR 32; 485160 results.
34. EMBASE; (orthopedic* OR orthopaedic*).ti,ab; 72499 results.
35. EMBASE; exp ORTHOPEDIC SURGERY/; 324212 results.
36. EMBASE; arthroplast*.ti,ab; 42593 results.
37. EMBASE; exp ARTHROPLASTY/; 50337 results.
38. EMBASE; ((total AND (hip OR knee OR ankle) AND replacement) OR thr OR tkr OR tar).ti,ab; 42927 results.
39. EMBASE; exp JOINT PROSTHESIS/; 46658 results.
40. EMBASE; (joint AND replacement).ti,ab; 12516 results.
41. EMBASE; 34 OR 35 OR 36 OR 37 OR 38 OR 39 OR 40; 422569 results.
42. EMBASE; 28 AND 33 AND 41; 233 results.
43. EMBASE; Duplicate filtered: [28 AND 33 AND 41]; 233 results.

Section 2 & 3. Modified Colman Scoring

Table S1. The Agreed Modified Coleman for the Generality of the article.

Study Size (n)	Criteria	Score
	>60	25
	41-60	20
	20-40	10
	<20	0
Mean follow-up (months)		
	>24	20
	12-24	10
	<12	5
Type of study		
	Randomised Control Trial	25
	Prospective cohort	15
	Retrospective cohort	5
Subject selection		
	Selection criteria reported and unbiased	10
	Recruitment rate reported	10
	All those eligible but not included accounted for	10
**Total Score**		**100**

Table S2. The agreed Modified Coleman weighted for areas of study interest.

**Assessment and severity grading**			
	Validated Score		50
	Invasive versus non-invasive		
		Non-Invasive	15
		Invasive	10
	Examination		
		Based on Signs	10
		Based on Symptoms	10
	Compliance		
		>80%	15
		60-80%	5
		<60%	0
	Assessor blinded		10
**Total score**			100
**Incidence**			
	Longitudinal follow-up		30
	Compliance follow-up		
		>80%	10
		50–80%	5
		<50%	0
	Duration of follow-up		
		>24 months	20
		12–24 months	10
		<12 months	5
	Type of study		
		Randomised Control Trial	30
		Prospective	15
		Retrospective	0
**Total score**			**90**

Table S3. Modified Coleman score summary, consideration by areas of interest.

	Articles	Generality	Proposed Definition	Assessment	Severity	Incidence
1	Brandjes et al, 1997 [7]	100	-	75	70	80
2	Kahn et al, 2009 [11]	-	Included	-	-	-
3	Beebe et al, 1996 [14]	-	Included	-	-	-
4	Eklof et al, 2004 [15]	-	Included	-	-	-
5	Kakkar et al, 1985 [16]	-	Included	-	-	-
6	Porter et al, 1995 [10]	-	Included	-	-	-
7	Porter et al, 1988 [17]	-	Included	-	-	-
8	Villalta et al, 1994 [18]	-	Included	-	-	-
9	Haenan et al, 2002 [20]	90	-	90	70	80
10	Kahn et al, 2011 [21]	65	-	90	80	0
11	Deehan et al, 2001 [22]	50	-	30	0	100
12	Schindler et al, 2005 [23]	70	-	65	50	90
13	McNally et al, 1994 [24]	75	-	80	60	100
14	Ginsberg et al, 2000 [25]	50	-	50	20	100
15	Mant et al, 2008 [26]	80	-	65	50	100
16	Delluc et al, 2010 [27]	80	-	65	70	80
17	Hach-Wunderle et al, 2013 [28]	70	-	65	70	80
18	Tick et al, 2010 [29]	90	-	80	60	80
19	Lonner et al, 2006 [30]	70	-	65	40	100
20	Kurtoglu et al, 2010 [31]	80	-	65	50	80
21	Ziegler et al, 2001 [32]	80	-	75	70	80
22	Kahn et al, 2014 [33]	100	-	90	80	80
23	Partsch et al, 2004 [34]	85	-	90	80	80
24	Kahn et al, 2005 [35]	90	-	75	70	80
25	Saarinen et al, 2002 [36]	75	-	80	70	80
26	Galanaud et al, 2012 [37]	90	-	65	70	80
27	Prandoni et al, 2004 [38]	100	-	75	70	80
28	Monreal et al, 1993 [39]	90	-	65	60	80
29	Fitzgerald et al, 2011 [40]	45	-	75	50	90
30	Sang et al, 2014 [41]	80	-	80	70	80
31	Ginsberg et al, 2001 [42]	90	-	75	65	80
32	McAndrew et al, 2010 [43]	65	-	35	10	90
33	Tick et al, 2008 [44]	75	-	65	60	70
